# Supplementary material for: Smooth Interpolating Curves with Local Control and Monotone Alternating Curvature
Source: Comput Graph Forum. 2022 Oct 6;41(5):25–38. doi: 10.1111/cgf.14600 (PMC9827861; doi:10.1111/cgf.14600)
Supplement: Supplementary file 1 — Supplement Material [file CGF-41-25-s001.zip › Local-Smooth-Interpolating-MonoCurvature/extern/clothoids/submodules/Utils/docs/api-cpp/function_a00082_1ad4adadc1f5136ae37fa104a17f717f33.html]

Function Utils::info — Utils v1.2

### Navigation

- index
- toc
- next
- previous
- Utils »
- C++ API »
- Function Utils::info

# Function Utils::info¶

- Defined in File Utils\_CPU\_info.hh

## Function Documentation¶

void Utils::info(bool &bMMX, bool &bMMXplus, bool &bSSE, bool &bSSE2, bool &bSSE3, bool &bSSSE3, bool &bSSE41, bool &bSSE42, bool &bSSE4a, bool &bSSE5, bool &b3Dnow, bool &b3DnowExt)¶
:   Get info of Intel CPU

    Parameters
    :   - **bMMX** – **[out]** booelan, support MMX instruction set
        - **bMMXplus** – **[out]** booelan, support MMXplus instruction set
        - **bSSE** – **[out]** booelan, support SSE instruction set
        - **bSSE2** – **[out]** booelan, support SSE2 instruction set
        - **bSSE3** – **[out]** booelan, support SSE3 instruction set
        - **bSSSE3** – **[out]** booelan, support SSEE3 instruction set
        - **bSSE41** – **[out]** booelan, support SSE41 instruction set
        - **bSSE42** – **[out]** booelan, support SSE42 instruction set
        - **bSSE4a** – **[out]** booelan, support SSE4a instruction set
        - **bSSE5** – **[out]** booelan, support SSE5 instruction set
        - **b3Dnow** – **[out]** booelan, support 3Dnow instruction set
        - **b3DnowExt** – **[out]** booelan, support 3DnowExt instruction set

### Quick search

### Table of Contents

- rang - Colors for your Terminal
- Zstr
- C++ API

«
hide menu

menu
sidebar
»

### Navigation

- index
- toc
- next
- previous
- Utils »
- C++ API »
- Function Utils::info

© Copyright 2021, Enrico Bertolazzi.
Created using Sphinx 4.2.0.
